# Anti-neoplastic properties of hydralazine in prostate cancer

**DOI:** 10.18632/oncotarget.1909

**Published:** 2014-04-17

**Authors:** Inês Graça, Elsa J Sousa, Pedro Costa-Pinheiro, Filipa Q Vieira, Jorge Torres-Ferreira, Maria Gabriela Martins, Rui Henrique, Carmen Jerónimo

**Affiliations:** ^1^ Cancer Biology & Epigenetics Group, Research Center of the Portuguese Oncology Institute-Porto; ^2^ Department of Pathology, Portuguese Oncology Institute-Porto; ^3^ Department of Hematology - Laboratory of Flow Cytometry, Portuguese Oncology Institute-Porto; ^4^ Departments of School of Allied Health Sciences ESTSP, Polytechnic of Porto; ^5^ Department of Pathology and Molecular Immunology, Institute of Biomedical Sciences Abel Salazar, University of Porto

**Keywords:** Prostate Cancer, Hydralazine, DNA methyltransferases, Androgen Receptor

## Abstract

Prostate cancer (PCa) is a major cause of cancer-related morbidity and mortality worldwide. Although early disease is often efficiently managed therapeutically, available options for advanced disease are mostly ineffective. Aberrant DNA methylation associated with gene-silencing of cancer-related genes is a common feature of PCa. Therefore, DNA methylation inhibitors might constitute an attractive alternative therapy. Herein, we evaluated the anti-cancer properties of hydralazine, a non-nucleoside DNA methyltransferases (DNMT) inhibitor, in PCa cell lines. *In vitro* assays showed that hydralazine exposure led to a significant dose and time dependent growth inhibition, increased apoptotic rate and decreased invasiveness. Furthermore, it also induced cell cycle arrest and DNA damage. These phenotypic effects were particularly prominent in DU145 cells. Following hydralazine exposure, decreased levels of *DNMT1, DNMT3a* and *DNMT3b* mRNA and DNMT1 protein were depicted. Moreover, a significant decrease in *GSTP1, BCL2* and *CCND2* promoter methylation levels, with concomitant transcript re-expression, was also observed. Interestingly, hydralazine restored androgen receptor expression, with upregulation of its target p21 in DU145 cell line. Protein array analysis suggested that blockage of EGF receptor signaling pathway is likely to be the main mechanism of hydralazine action in DU145 cells. Our data demonstrate that hydralazine attenuated the malignant phenotype of PCa cells, and might constitute a useful therapeutic tool.

## INTRODUCTION

Prostate cancer (PCa) is the second most common malignant neoplasm diagnosed in men and the sixth leading cause of cancer-related mortality worldwide [1]. Although most early-diagnosed patients have optimal survival rates after treatment with prostatectomy or radiotherapy, treatment of advanced disease is mainly ineffective and remains a clinical challenge [2]. Androgen-deprivation therapy, by pharmacological or surgical castration, is widely used for locally advanced and systemically spread PCa. However, most patients that initially respond to this type of therapy have a median time for progression to castration-resistant disease of only 18-30 months [3]. Therefore, investigation of new and more effective therapeutic strategies is urgently needed for this important subset of PCa patients. Alterations in DNA methylation are early events in human carcinogenesis, including PCa, which are conserved during cancer progression [4]. Aberrant methylation of CpG dinucleotides in gene promoter regions is associated with transcriptional repression, not only of tumor-suppressor genes, but also of genes with important regulatory functions [5, 6]. DNA methyltransferases (DNMTs) are a group of enzymes responsible for the establishment and maintenance of methylation patterns. Because epigenetic alterations, contrarily to genetic modifications, are reversible, inhibition of DNMTs might have the ability to overturn tumor cell phenotype, constituting an attractive therapeutic target [7]. So far only two nucleoside analogues, 5-azacytidine (5-Aza-CR) and 5-aza-2′-deoxycytidine (5-Aza-CdR) have been approved by Food and Drug Administration (FDA) for clinical use, specifically in myelodysplastic syndrome [8]. Nevertheless, their clinical effectiveness is not entirely dependent on their DNA methylation inhibitory activity and its efficacy in solid tumors is yet to be fully demonstrated [9]. Indeed, 5-Aza-CdR was shown to have modest clinical activity against castration-resistant PCa in a phase II clinical trial [10]. Due to the toxicity of nucleoside analogues, efforts have been made to develop and test non-nucleoside compounds that target DNMTs, which are not incorporated in the DNA and/or RNA, for solid tumors. Recently, we reported RG108 as an effective tumor growth suppressor in PCa cell lines. However, this drug is substantially more effective in androgen-responsive than in castration-resistant cell lines [11]. Among non-nucleoside analogues, hydralazine, a potent arterial vasodilator approved by FDA for treatment of severe hypertension, heart failure and hypertension in pregnancy, has been described as a weak non-nucleoside DNA methylation inhibitor [12-14]. Furthermore, this drug has been shown to demethylate and reactivate the expression of several cancer-related genes and its activity is synergistic with that of the histone deacetylase inhibitor (HDACi) valproic acid, either *in vitro* or *in vivo* [15, 16]. In patients carrying solid tumors refractory to conventional treatment, clinical trials have been conducted in which those epigenetic drugs were combined with conventional therapy. It was shown that this regimen was not only well tolerated, but, importantly, it overcome tumors' chemotherapy resistance and induced radiosensitivity [17, 18]. To the best of our knowledge, the antineoplastic effect of hydralazine in PCa has not been previously investigated. Therefore, in the context of a broader research project intended to determine the therapeutic efficacy of compounds targeting epigenetic alterations of PCa cells, we aimed at evaluating the impact of hydralazine as a PCa growth inhibitor as well as its effect on DNA demethylation activity and consequent reactivation of genes known to be epigenetically silenced in this neoplasm. Additionally, we investigated the cellular pathway through which hydralazine exerts its growth-inhibitory effect. We found that hydralazine was able to reverse PCa cell phenotype, decrease DNMTs expression and gene promoter methylation with concomitant expression's restoration of silenced genes involved in prostate carcinogenesis. Moreover, this compound was capable of restoring androgen receptor (AR) expression in DU145 cell line. Importantly, we found that hydralazine growth-inhibitory effects occur in cell cycle and EGF receptor signaling pathway inhibition.

## RESULTS

### Hydralazine reverts PCa cells malignant phenotype

**Figure 1:**
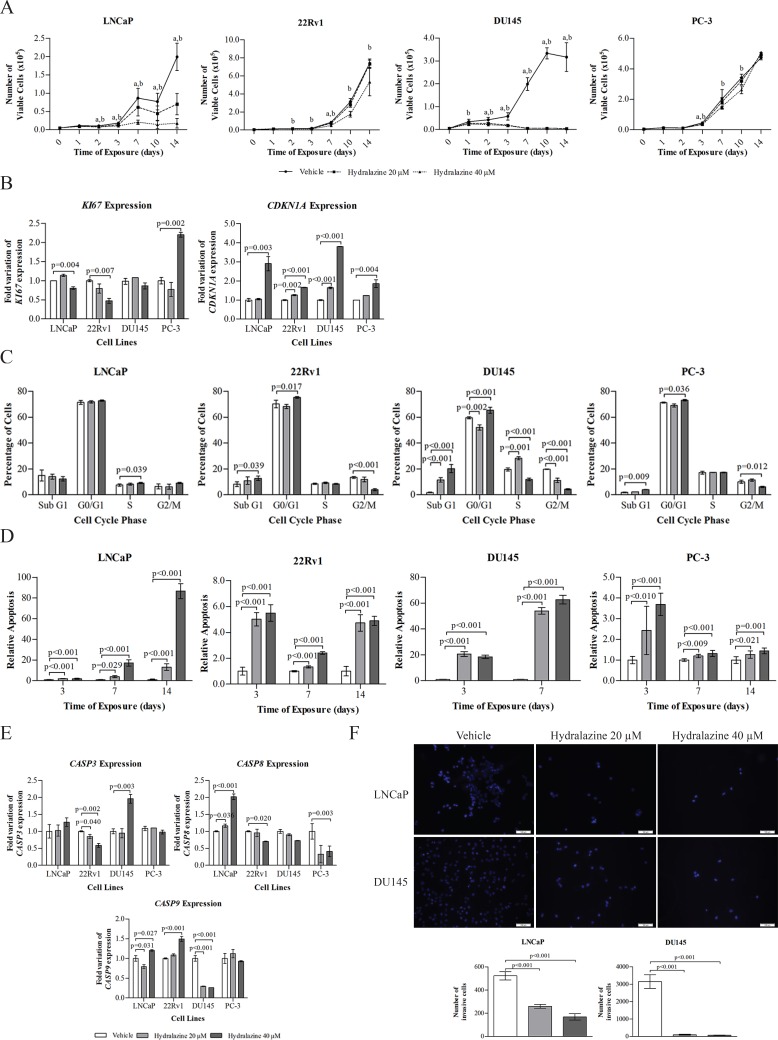
Phenotypic effects induced by hydralazine in PCa cell lines (A) Cell viability in LNCaP, 22Rv1, PC-3 and DU145, exposed to hydralazine and drug vehicle, at days 0, 1, 2, 3,7,10, and 14, measured by MTT assay. (a) Statistically significant differences were observed between vehicle and 20 μM hydralazine, and (b) vehicle and 40 μM hydralazine. (B) mRNA expression of *Ki67* and *CDKN1A* in LNCaP, 22Rv1 and PC-3 after 14 days and DU145 after 3 days of exposure to hydralazine. (C) Cell cycle evaluation after 14 days of hydralazine exposure in LNCaP, 22Rv1 and PC-3 and three days of hydralazine exposure in DU145. (D) Effect of hydralazine exposure in early apoptosis of PCa cell lines, measured at days 3 and 7 in all PCa cell lines and at day 14 in LNCaP, 22Rv1 and PC-3, with a phosphatidylserine based assay. (E) *CASP3, CASP8* and *CASP9* mRNA expression in LNCaP, 22Rv1 and PC-3 after 14 days and DU145 after 3 days of exposure to hydralazine. (F) Effect of hydralazine exposure on the invasion potential of LNCaP and DU145 (upper – immunofluorescence images of vehicle and hydralazine exposed cells, counterstained with DAPI; down – graphic representation of the total number of invasive cells). All data are presented as mean of three independent experiments ± s.d.

The half-maximal effective concentration (EC50) of hydralazine was calculated in two PCa cell lines (LNCaP and DU145) after 72 hours of drug exposure. The drug displayed an EC50 of 63 μM in LNCaP and 30 μM in DU145 (Supplementary Figure 1). To investigate the effects of hydralazine on the malignant phenotype of PCa, four human PCa cell lines (LNCaP, 22Rv1, DU145, and PC-3) were exposed to two different concentrations of this drug (20 and 40 μM) or to the vehicle (PBS) during 14 days, as previously described for RG108 [11]. Cell viability was evaluated at days 0, 1, 2, 3, 7, 10, and 14. Exposure to hydralazine markedly reduced cell viability, especially at 40 μM concentration (Fig. 1A). Remarkably, a significant decrease in the number of viable cells was observed at the day 2, with a more pronounced effect after 14 days of exposure to both drug concentrations in LNCaP, whereas for 22Rv1 a less impressive effect was observed, even after exposure to the highest concentration. PC-3 treated cells also depicted a slight reduction at the end of day 3 through day 10. Interestingly, DU145 was the most sensitive cell line, since inhibition of cell viability was achieved in all tested days for both drug concentrations. These results were corroborated by mRNA expression levels of two genes involved in cell proliferation. Due to its low viability rate, this cell line was evaluated after three days of drug exposure. Indeed, a significant induction of *CDKN1A* and decrease of *Ki67* mRNA levels was observed in hydralazine treated cells compared to the respective vehicle (Fig. 1B). Additionally, alterations in cell cycle distribution were evaluated and a significant cell cycle arrest was observed at G0/G1 for all cell lines, except for LNCaP in which the arrest was observed at S phase (Fig. 1C). A significant increase in apoptosis was depicted for all cell lines with both drug concentrations (Fig. 1D). To confirm the activation of the apoptotic pathway, *CASP8, CASP9,* and *CASP3* mRNA levels were also evaluated. A statistically significant increase in transcript levels of *CASP3* and *CASP8* was found for DU145, and LNCaP, respectively, whereas *CASP9* expression levels were increased in LNCaP and 22Rv1 cells (Fig. 1E). Furthermore, a significant increase in Sub-G1 cell population was observed for three of the cell lines [22Rv1, DU145 and PC-3 (Fig. 1C)]. The two best responsive PCa cell lines, DU145 and LNCaP, also showed a significant decrease in invasion ability after 48 hours exposure to hydralazine (Fig. 1F).

### Hydralazine disrupts the expression of cell cycle genes

**Figure 2:**
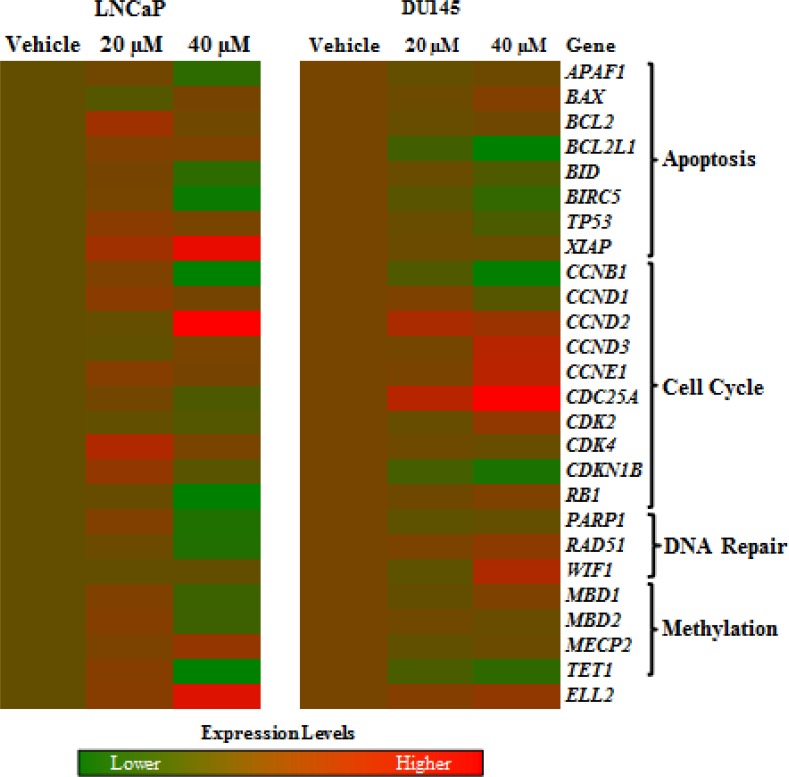
Effects of hydralazine in cellular pathways Image of deregulated genes after exposure of LNCaP (left) and DU145 (right) to 20 and 40 μM hydralazine. Induced in gene expression is represented in red, while decreased expression is shown in green. Real-time RT-PCR values were normalized to three reference genes (*18S, TFRC* and *GUSB*). All experiments were performed in triplicate.

A panel of genes representative of critical cellular pathways were selected for assessment of expression in LNCaP and DU145 (Fig. 2). Globally, cell cycle-associated genes were upregulated and comparative analysis between the most altered genes in two cell lines, allowed for the identification of five upregulated (*CCND2, ELL2, BAX, CCND3* and *CCNE1*) and five downregulated (*BIRC5, PARP1, CCNB1, BID* and *TET1*) genes.

### Hydralazine induce DNA Damage in PCa Cell Lines

**Figure 3:**
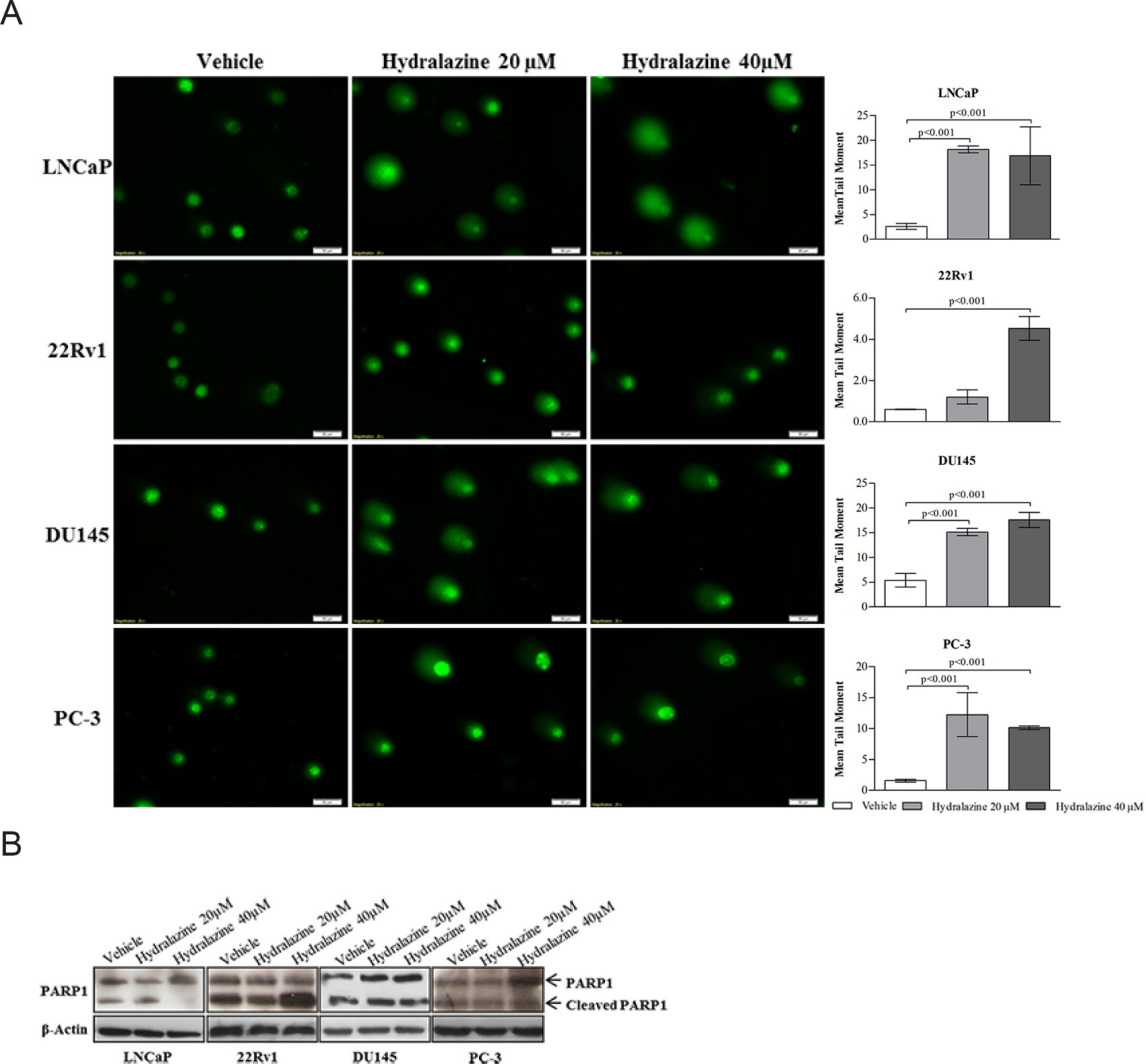
Hydralazine effect on DNA damage (A) Left - Comet assay immunofluorescence images of vehicle and hydralazine exposed cells counterstained with Syber Green and right - graphic representation of mean tail moment. (B) PARP1 protein expression analyzed by western blot. Full length PARP1 is represented by a band of 116 kDa and cleaved PARP1 is represented by a 89 kDa band. β-Actin was used as a loading control. All data are presented as mean of three independent experiments ± s.d.

PCa cells exposed to hydralazine showed significant DNA damage compared to those exposed to the vehicle, as depicted by the increase in tail length of the comet (Fig. 3A). Remarkably, an increase in protein expression of cleaved PARP1, a protein involved in DNA repair, was found in 22RV1 whereas a decrease was displayed by LNCaP. No changes in expression of this protein were observed in DU145 and PC-3 cells (Fig. 3B).

### Hydralazine decreases DNMTs expression and leads to demethylation and reactivation of silenced genes in human PCa cells

**Figure 4:**
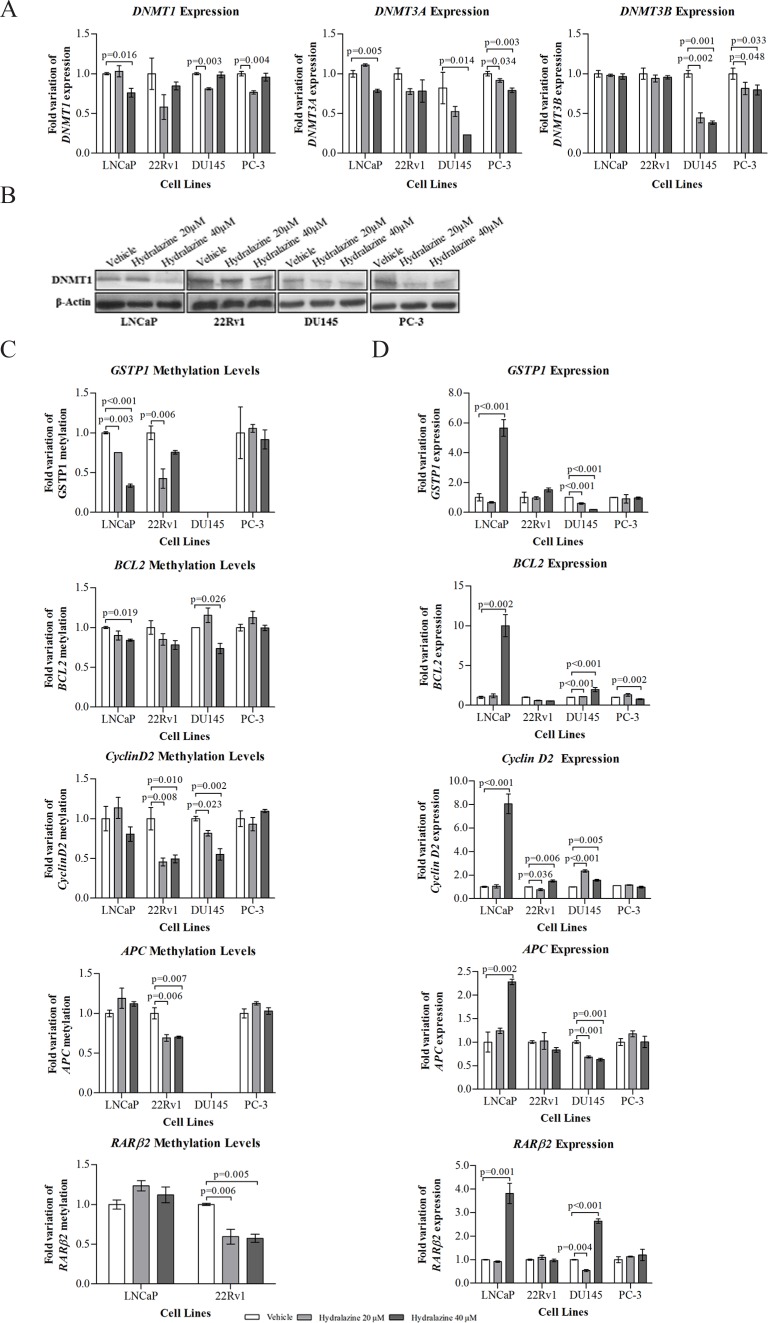
Impact of hydralazine on specific gene methylation of PCa cells (A) Real-time RT-PCR of mRNA levels of *DNMT1, DNMT3a* and *DNMT3b* normalized to *GUSB* in vehicle and drug exposed cells. (B) Western blot analysis of DNMT1 protein expression. β-Actin was used as a loading control. (C) Real-time methylation-specific PCR (qMSP) for assessment of *GSTP1, APC, RARβ2, CCND2* and *BCL2* methylation levels, normalized to *β-Actin*. (D) mRNA expression of *GSTP1, APC, RARβ2, CCND2* and *BCL2,* by real-time RT-PCR, in both hydralazine and vehicle exposed cells. All data are presented as mean of three independent experiments ± s.d.

A reduction in mRNA expression for all three DNMTs was depicted in DU145 and PC-3 cells. A significant decrease in *DNMT1* was achieved after exposure to 20 μM hydralazine in *DNMT3a*, after 40 μM in DU145 and with both drug concentrations in PC-3; as well as for *DNMT3b* with both concentrations in both cell lines. LNCaP only showed decreased levels of *DNMT1* and *DNMT3a* when exposed to 40 μM hydralazine (Fig. 4A). Notably, a reduction in DNMT1 protein expression was observed for all tested cell lines (Fig. 4B). Gene promoter methylation status and expression levels of five genes (*GSTP1, APC, RARβ2, CCND2* and *BCL2*), previously reported to be silenced by promoter hypermethylation in PCa, was evaluated. Both LNCaP and 22Rv1 showed a significant decrease in methylation levels of *GSTP1* with 20 μM whereas only LNCaP exhibited similar results with 40 μM (Fig. 4C). Despite both cell lines presented a trend for increase *GSTP1* mRNA expression when exposed to 40 μM hydralazine, only in LNCaP statistical significance was observed (Fig. 4D). DU145 cells did not show significant *GSTP1* promoter methylation. Concerning *APC* and *RARβ2,* only 22Rv1 demonstrated a significant decrease in promoter methylation levels (Fig. 4C). Interestingly, significant re-expression of those genes was only achieved in LNCaP and DU145 (Fig. 4D). *BCL2* methylation levels were reduced in LNCaP and DU145 (Fig. 4C) but mRNA re-expression was observed following exposure to 40 μM hydralazine (Fig. 4D). Although a significant reduction in *CCND2* methylation was observed only in 22Rv1 and DU145 with both drug concentrations (Fig. 4D), gene re-expression was also achieved in LNCaP (Fig. 4D).

### Hydralazine induces histone acetylation in LNCaP and DU145 cell lines

**Figure 5:**
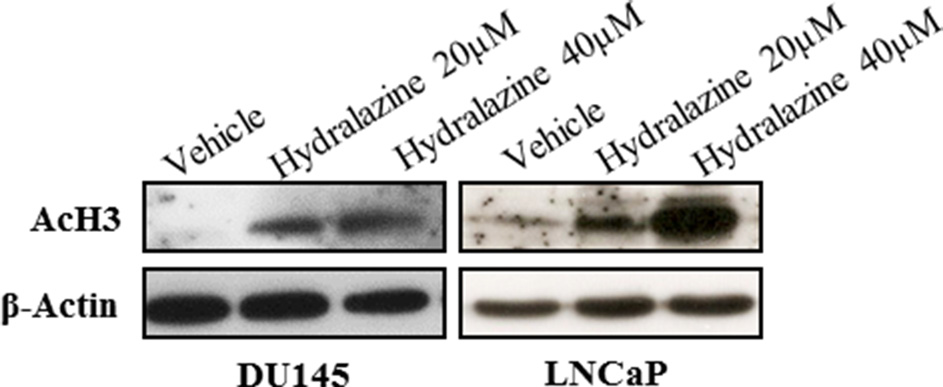
Effect of hydralazine on histone 3 acetylation (AcH3) Western Blot analysis of AcH3, in LNCaP (after 14 days of drug exposure) and DU145 (after 3 days of drug exposure). β-Actin was used as a loading control.

Since hydralazine induced re-expression of *CCND2, RARβ2* and *APC* in LNCaP and *RARβ2* in DU145, without a concomitant significant reduction in DNA methylation, and because histone acetylation interplays with DNA methylation, the acetylation of Histone 3 (AcH3) was evaluated by western blot in those two cell lines. Interestingly, exposure to both concentrations of hydralazine increased AcH3 expression in LNCaP and DU145 (Fig. 5). In addition, histone methyltransferase G9A expression was also evaluated, but no significant differences were found between vehicle and hydralazine treated cells (Supplementary Figure 2).

### Hydralazine restores Androgen receptor expression in DU145 cell line

**Figure 6:**
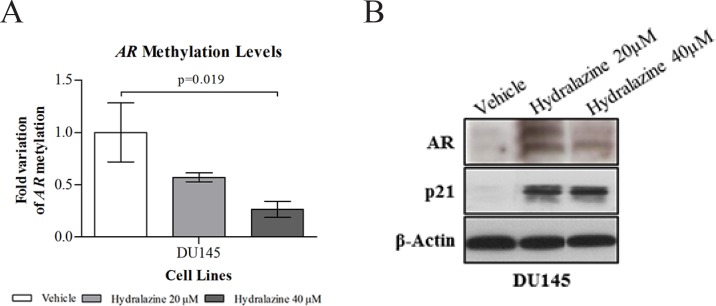
Reactivation of androgen receptor (AR) expression upon exposure to hydralazine (A) Promoter methylation levels of AR in DU145 cell line assessed by real-time methylation-specific PCR (qMSP) after 3 days of drug exposure. AR methylation levels were normalized to β-Actin. Data are presented as mean of three independent experiments ± s.d. (B) AR and p21 protein expression determined by western blot, after 3 days of hydralazine exposure. β-Actin was used as loading control.

It is widely acknowledged that one of the mechanisms of androgen receptor (AR) silencing in castration-resistant cell line DU145 is promoter hypermethylation [19]. After three days of exposure to 40 μM of hydralazine a significant reduction in *AR* methylation levels was observed (Fig. 6A) with concomitant AR protein re-expression, at both drug concentrations (Fig. 6B). Re-expression was more evident when cells were exposed to 20 μM hydralazine. To further demonstrate AR activation, an increase in p21 protein expression was detected after drug exposure (Fig. 6B), which is in accordance with the reported ability of AR to upregulate p21 in PCa cell lines [20].

### Hydralazine may exert its growth-inhibitory effect in DU145 cells by interfering with EGF signaling pathway

**Figure 7:**
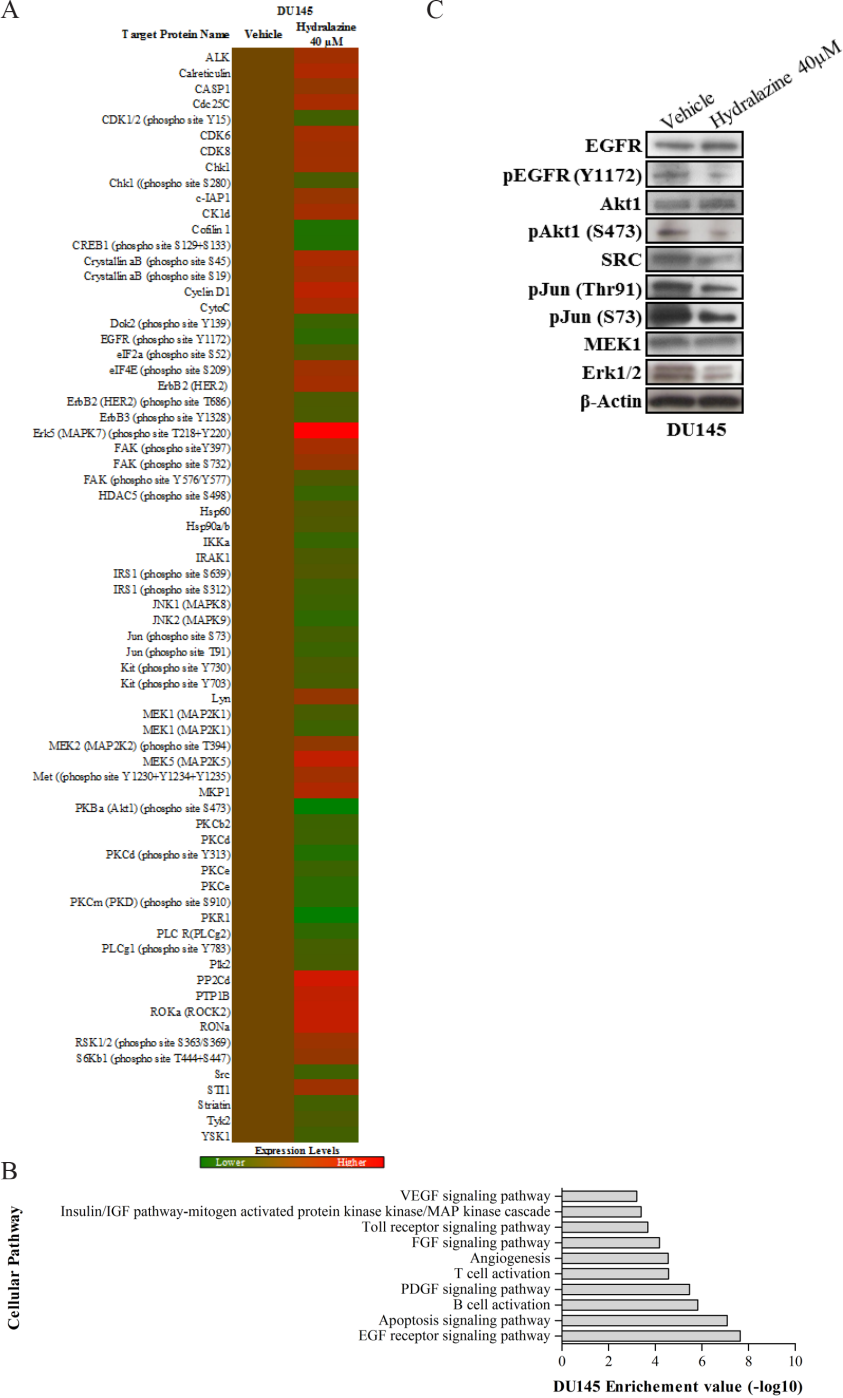
EGF signaling pathway deregulation in DU145 cells after hydralazine exposure (A) Protein expression heat map of DU145exposed to 40 μM hydralazine (red: protein overexpression; green: protein reduced expression. Experiments were performed in triplicate. (B) Altered cellular pathways identified by DAVID software. EGF receptor pathway showed the lowest p value (10^−8^). (C) Western Blot validation of disrupted targets of EGF receptor pathway in DU145 cell line upon hydralazine exposure. β-Actin was used as loading control

Because DU145 showed the largest sensitivity to hydralazine, it was chosen to investigate the cellular mechanisms and pathways underlying these pronounced effects, using a protein array. After drug exposure, 69 proteins presented altered expression, of which 40 were considered downregulated and 29 upregulated (Fig. 7A). These 69 proteins were then analyzed with DAVID (Database for Annotation, Visualization and Integrated Discovery) and several cellular networks were identified as being altered after drug exposure (Fig. 7B). However, EGF receptor signaling pathway (p-value = 1×10^−8^) scored the highest significance value. Therefore, several of the identified underexpressed members in array platform were further validated (EGFR, EGFR phosphorylated at Y1172, Akt1, phosphorylated Akt1 at S473, SRC, phosphorylated C-Jun at Thr91 and S73, MEK1 and ERK1/2) by Western blot analysis (Fig. 7C). Moreover, EGFR was reported to be oncogenic activated by somatic mutations in PCa [21]. In order to ascertain if the EGFR pathway was intact in PCa cell lines, we sequenced EGFR in LNCaP, 22Rv1, DU145 and PC-3 cells for mutational hotspots regions. No EGFR mutations were found in any of the four tested PCa cell lines.

## DISCUSSION

Although most diagnosed PCa remain localized, hardly threatening life expectancy, about one third of those tumors metastasize to distant organs and eventually cause patient death. Median survival for patients with localized PCa is more than five years compared with one to three years for patients with metastatic disease. Although first line therapy for metastatic PCa is either pharmacological or surgical castration, most patients become androgen refractory within a relatively short period of time. Available therapies for castration-resistant PCa only aim to reduce symptoms and improve in overall survival is only about two months [2]. Therefore, new therapeutic options for this disease stage are mandatory. Cancer-related gene promoter methylation is an emerging molecular marker in PCa and at least 100 genes involved in several cellular functions are *de novo* methylated during prostate carcinogenesis [22]. Therefore, DNMTi might be a valuable therapeutic tool for PCa. The two FDA approved nucleoside DNMTi, however, are of limited effectiveness in solid tumors, including PCa [9, 10]. This might be due to limited incorporation into PCa cells, which are much less proliferative, compared to hematological malignancies. This might be overcome with the use of non-nucleoside DNMTi, which are independent of replication for incorporation into DNA.

Herein, we report, for the first time, that hydralazine, a non-nucleoside DNMTi, has anti-tumoral effect in PCa cells. This compound reduced cell viability and induced cell death by apoptosis in PCa cell lines in a dose and time-dependent manner, being 40 μM the best drug concentration in all tested cell lines. Similar results were observed for human cervical cancer cells when exposed to 40 μM, resulting in 40 to 50% of cell growth inhibition and induction of apoptosis, without affecting normal cells [15]. Importantly, both inhibition of cell viability and apoptosis induction were confirmed at molecular level, the former by a significant decrease in the cell proliferation marker *Ki67* and an increase in *CDKN1A*, a gene that codifies p21 a well-known cell cycle inhibitor, and the latter by induction of caspases' expression. Furthermore, our data also show that hydralazine induces cell cycle arrest at G0/G1 in three PCa cell lines, which is in accordance with a previous report in bladder cancer [23]. Moreover, LNCaP cells also demonstrated cell cycle arrest at S phase after hydralazine exposure, similar to human cervical cancer cells [15]. Importantly, hydralazine drastically reduced the invasive potential of PCa cells, a feature that is particularly relevant because metastatic spread is a major cause of PCa morbidity and mortality.

Exposure of PCa cell lines to hydralazine resulted in significant induction of DNA damage, accompanied by significant PARP-1 decrease in LNCaP cells. Interestingly, previous studies have shown that hydralazine exposure induced both DNA cleavage and damage [24, 25]. The lack of efficient DNA repair is often an advantage for cancer therapy (especially radio- and chemotherapy), based on the concept of synthetic lethality, which states that cancer cells are more proliferative and thus more susceptible to induced DNA damage than normal cells. In fact, DNA repair is considered a mechanism of cancer-therapy resistance [26]. Currently, several agents capable of inhibiting PARP-1 are under test and those drugs might be useful as chemosensitizers for cancer therapy [27]. Taking in consideration our results is tempting to speculate whether hydralazine might be useful as a chemosensitizer, acting in concert with conventional therapeutic strategies.

Remarkably, hydralazine was not only able to inhibit *DNMT1, 3a* and *3b* mRNA expression in three of the four tested cell lines, but also DNMT1 protein expression in all cell lines. Moreover, our data indicate that the reduction in DNMT1 expression might not be dependent on its regulator and co-factor G9A, since no alterations were observed for G9A expression after drug exposure. The ability of hydralazine to reduce DNMTs expression had already been described in other tumor models [28, 29]. Several studies demonstrated that DNMT1 is overexpressed in human PCa tissues, compared to normal prostate, leading to deleterious inactivation of tumor-suppressor genes and might be associated with tumor progression and poor prognosis [30]. Therefore, the reduction of DNMTs expression might be related with the observed reversal of *GSTP1, BCL2* and *CCND2* promoter hypermethylation with concomitant re-expression upon hydralazine exposure. Interestingly, hydralazine's ability to demethylate the promoter region of tumor-suppressor genes, including *GSTP1, RARβ2* and *p16,* causing its re-expression has been documented in several cancers [23, 29]. Nonetheless, for *CCND2, RARβ2* and *APC*, gene re-expression was achieved without significant promoter demethylation, in our study. Concerning the *AR* promoter, a significant reduction in methylation levels was only observed with the highest concentration of hydralazine, although re-expression was already apparent at the lowest concentration. These findings further demonstrate that epigenetic silencing of cancer-related genes is a complex phenomenon, in which other mechanisms, such as histone post-translational modifications are also implicated, in addition to promoter methylation [22]. Thus, the observed increase in AcH3 following hydralazine exposure might have contributed to *CCND2* and *AR* re-expression.

As previously stated, hydralazine restored *AR* mRNA and protein expression in DU145 cells, in which *AR* is silenced due to promoter hypermethylation [19]. Similar results were reported for estrogen receptor (ER) protein in the ER negative MDA-231 breast cancer cell line [23]. Interestingly, AR re-expression has been also achieved following chronic exposure (20 days) to 5-Aza-CR, resulting in a marked decrease of tumor cell proliferation and a significant increase of AR and PSA protein levels [31]. The lack of AR expression has been associated with increased invasive properties, suggesting that therapies able to restore its expression receptor may reduce tumor dissemination [32]. Therefore, the restoration of AR expression by hydralazine might be associated with the observed reduction in the invasive potential of DU145 cells, and could also justify the remarkable decrease in cell proliferation by the upregulation of its inhibitor, p21. Hence, it is suggested that patients with castration-resistant PCa might become more responsive to hormone therapy following hydralazine administration, thus slowing PCa progression. Remarkably, the combination of 5-Aza-CdR with the anti-androgen bicalutamide demonstrated a synergistic effect, increasing the susceptibility of PCa cells to undergo apoptosis and repressing tumor growth in xenograft mice [31, 33]. Recently, high expression levels of DNMT3a and DNMT3b have been associated with bicalutamide resistance and, consequently, with the emergence of the castration-resistant phenotype [34]. Because hydralazine was shown to decrease all DNMTs expression in DU145, a synergistic action between this drug and conventional anti-androgenic therapy is likely to exist.

**Figure 8:**
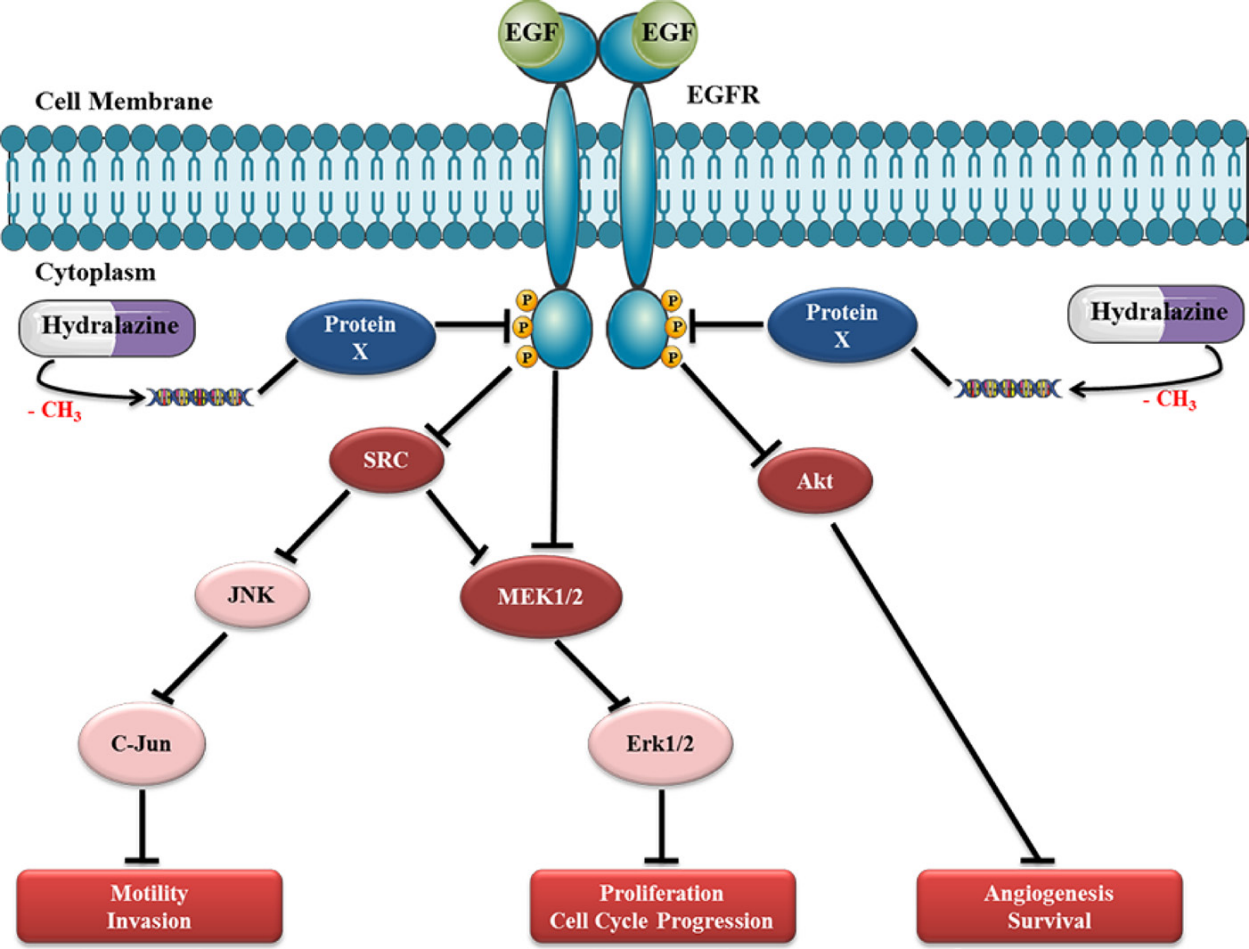
Proposed model for hydralazine disruption of EGF receptor pathway in DU145 cell line Exposure of PCa cells to hydralazine probably leads to demethylation of a critical gene that codifies a protein X that regulates EGFR phosphorylation. The re-expression of this protein will induce a significant decrease in EGFR phosphorylation and, consequently, in downstream targets, namely, SRC, MEK1/2 and Akt. Reduced expression of SRC may cause a decrease in JNK and C-Jun proto-oncogene leading to decreased motility and invasion capacity of PCa cells. Moreover, a decrease in MAPK pathway induced by SRC and/or directly via EFGR impairment, could explain the decrease in proliferation and cell cycle arrest observed in this cell line after hydralazine exposure. Finally, a decrease in Akt expression may lead to cell death and decreased tumor angiogenesis. The disruption of these cancer networks through deregulation EGF pathway by hydralazine might be responsible for the attenuation of the malignant phenotype of DU145 cells.

DU145 cell line was shown to be highly responsive to hydralazine because unlike others cell lines, which required 14 days of drug exposure to achieve pronounced phenotypic effects, it demonstrated maximal anti-cancer effects upon only three days of exposure. Based on the analysis of a selected panel of 854 proteins we suggest that the remarkable effects of hydralazine on DU145 cells are mostly due to disruption of EGF receptor signaling pathway. EGFR is overexpressed in PCa and plays an important role in cancer cell growth, especially during androgen withdrawal [35]. Considering the important role of EGFR in prostate carcinogenesis, it has been suggested that a general inhibition of this pathway could provide therapeutic advantage against metastatic PCa [36]. Interestingly, negative regulators of EGFR phosphorylation (e.g., Syk) are known to be silenced by promoter methylation in PCa [37]. Therefore, we postulate that hydralazine may cause demethylation and consequent re-expression of those proteins, decreasing EGFR phosphorylation and activation. In support of this hypothesis, DU145 cell line exposed to hydralazine showed downregulation of all major downstream proteins in the EGF pathway (Fig. 8). Reduced expression of the phosphorylated form of EGFR might justify the decreased expression of its direct targets, Src, Akt and MEK1/2. Src has been described to be highly expressed in PCa, which may lead to JNK and C-Jun activation and, subsequently, to an increase in motility and invasion potential of PCa cells. Furthermore, it may activate MEK1/2 and, consequently, Erk1/2, increasing cell proliferation [38]. This MAP kinase pathway can also be directly activated by EGFR phosphorylation. Interestingly, the synergism between SRC and EGFR in PCa may be associated with a more aggressive tumor phenotype [39]. Moreover, Akt phosphorylation has been associated with PCa progression and it was suggested as an excellent biomarker for biochemical recurrence [40]. Importantly, through the interaction with EGF pathway, hydralazine may disrupt several cancer cell networks: by means of SRC, it may lead to a decrease in motility and invasion of tumor cells, through AKT, downregulate neoangiogenesis and increase apoptosis, and, finally, through, MAP kinase pathway, decrease tumor cell proliferation.

Because, hydralazine can maintain gene re-expression for longer periods of time, it was suggested that it has higher demethylase activity and, therefore, more effectiveness than 5-aza-CdR [14]. Thus, hydralazine might be more attractive from a clinical standpoint because to keep tumor-suppressor genes demethylated, cancer cells need to be chronically exposed to DNMTi. Because 5aza-CdR is incorporated into the DNA, a widespread loss of DNA methylation may occur, resulting in genome hypomethylation, which may ultimately predispose to neoplastic transformation of normal cells [41]. On the contrary, hydralazine inhibits DNA methylation by establishing highly stable interactions between its nitrogen atoms and DNMT active site. Moreover, hydralazine has been widely used as an anti-hypertensive agent, orally administered on a daily basis for long periods of time with minimal secondary effects. Thus, compared to FDA-approved 5-aza-CdR, hydralazine demonstrates a safer profile and might be more attractive for clinical use.

In summary, we showed that the non-nucleoside DNMTi hydralazine attenuates the PCa cell phenotype by disrupting several cancer cell pathways, in particular the EGF pathway in DU145 cells. Moreover, exposure to hydralazine decreased DNMTs expression and induced histone acetylation, which might be responsible for re-expression of genes known to be epigenetically silenced in PCa. Interestingly, hydralazine restored AR expression in DU145 cells, providing clues to a potential synergistic effect with conventional anti-androgenic therapy. *In vivo* studies are now mandatory to further validate the promising role of hydralazine in PCa therapy.

## MATERIALS AND METHODS

### PCa cell lines and drug preparation

PCa cell lines LNCaP and PC-3 were kindly provided by Prof. Ragnhild A. Lothe from the Department of Cancer Prevention at the Institute for Cancer Research, Oslo, Norway, whereas DU145 was kindly provided by Professor Fátima Baltazar at ICVS, University of Minho, Braga, Portugal and 22Rv1 cells were kindly provided by Dr. David Sidransky at the Johns Hopkins University School of Medicine, Baltimore, MD, USA. LNCaP and 22Rv1 cells were grown in RPMI 1640, DU145 cells were maintained in MEM and PC-3 cells were grown in 50% RPMI-50% F-12 medium (GIBCO, Invitrogen, Carlsbad, CA, USA). All basal culture media were supplemented with 10% fetal bovine serum and 1% penicillin/streptomycin (GIBCO, Invitrogen, Carlsbad, CA, USA). Cells were maintained in an incubator at 37ºC with 5% CO_2_. All PCa cell lines were karyotyped by G-banding (for validation purposes) and routinely tested for *Mycoplasma spp*. contamination (PCR Mycoplasma Detection Set, Clontech Laboratories). Hydralazine was purchased from Sigma (Sigma-Aldrich, St. Louis, MO, USA) dissolved in PBS (GIBCO, Invitrogen, Carlsbad, CA, USA). For control purposes, cell lines were exposed to the vehicle of the drug (PBS).

### Viability assay

Cell viability was evaluated by MTT assay. Briefly, PCa cells were seeded at 5 × 10^3^ cells/mL onto 96-well flat bottoned culture plates, allowed to adhere overnight and exposed to different Hydralazine concentrations (*i.e.*, 20 and 40 μM) for 14 days. At each time point 0.5 mg/ml of MTT reagent [3-(4, 5dimethylthiazol-2-yl)-2, 5-diphenyl-tetrazolium bromide] was added to each well, and the plates were incubated in the dark for 3 hours at 37ºC. Formazan crystals were then dissolved in DMSO and absorbance was read at 540 nm in a microplate reader (FLUOstar Omega, BMG Labtech, Offenburg, Germany), subtracting the background, at 630 nm. The number of cells was calculated using the formula: [(OD experiment × Number of cells at day 0) / Mean OD at day 0]. Three replicates were performed for each condition, and at least three independent experiments were carried out.

### Apoptosis evaluation

Evaluation of apoptosis was performed using APOPercentage apoptosis assay kit (Biocolor Ltd., Belfast, Northern Ireland) according to the manufacturer's instructions. PCa cells were seeded at 1 × 10^4^ cells/mL onto 24-well plates. Apoptotic cells were assessed at the end of the day 3, 7 and 14 for LNCaP and 22Rv1, 3 and 7 for LNCaP and 3 and 14 for PC-3, in a FLUOstar Omega microplate reader at 550 nm and the background subtracted at 620 nm. The results were normalized to number of viable cell obtained in the MTT assay according to the following formula (OD of apoptosis assay at day x/OD of MTT at day x).

### Cell cycle analysis

Cell cycle distribution of PCa cells was determined by flow cytometry. Briefly: 5×10^5^ harvested cells were fixed overnight at 4ºC with 70% cold ethanol. After washing with cold PBS, cells were ressuspended in staining Propidium Iodide Solution (Cytognos S.L, Salamanca, Spain) and incubated for 30 minutes at room temperature. All cells were then measured on a Cytomics FC500 flow cytometer (Beckman Coulter, Fullerton, CA, USA) and analyzed using Modfit LT (Verity Software House, Inc, Topshan, Maine, USA).

### Single Cell Gel Electrophoresis (Comet Assay)

After drug exposure, 50.000 cells were harvested by trypsinization, washed in PBS and re-suspended in 75 μl of low-melting point agarose (Invitrogen, Carlsbad, CA, USA). This suspension was then applied on top of the base layer consisting of normal-melting point agarose in a slide, after which it polymerized for 10 minutes at 4ºC. The slides were then immersed in lysis solution (2.5 M NaCl, 100 mM Na_2_EDTA, 10 mM Tris Base and 1% Triton X-100) at 4°C during 2 hours in the dark. To allow DNA to unwind, slides were posteriorly incubated in an alkaline electrophoresis buffer (300 mM NaOH, 1 mM Na2EDTA, pH 13) for 40 minutes at 4ºC. Electrophoresis was accomplished on a horizontal electrophoresis platform at 4ºC for 20 minutes at 15 V. Next, they were incubated in a neutralization buffer (Tris–HCl; pH-7.5) for 10 minutes. After fixation with ethanol 100% the slides were stained with Sybr Green^®^ (Life Technologies, Foster City, CA, USA) and DNA damage was evaluated under a fluorescent microscope. At least three independent experiments were performed for each condition. The DNA damaging effect in terms of DNA fragmentation was determined by measuring four parameters that included, tail moment, tail length, percentage of DNA in tail of the comet and 50 DNA-damaged cells were counted at least, for each condition.

### Cell Invasion Assay

Cell invasion was determined using BD BioCoat Matrigel Invasion Chamber (BD Biosciences, Franklin Lakes, NJ, USA). Briefly, 5×10^4^ cel/mL of LNCaP and DU145 cells were added to the upper chamber. Both cell lines were exposed to 20 and 40 μM of Hydralazine as well as to the vehicle for 48 hours, after which the non-invading cells were removed with cotton swabs from the upper side of the membrane. The membrane botton containing invading cells was fixed in methanol, washed in PBS and stained with DAPI (Vector Laboratories, Burlingame, CA). All the invading cells were counted under a fluorescent microscope. Three independent experiments were performed for each condition, and at least two experimental replicates were performed.

### Real Time Quantitative PCR (RT-qPCR)

After drug exposure RNA was extracted from cell lines using TRIzol® (Invitrogen, Carlsbad, CA, USA) according to manufacturer's instructions. First strand synthesis was performed using the high-capacity cDNA Reverse Transcription Kit from Applied Biosystems (Foster City, CA, USA). Expression of target genes (*Ki67, CDKN1A*, PARP1, *APC, RARβ2, GSTP1, CCND2, DNMT1, DNMT3a and DNMT3B*) was quantified using Taqman expression assays, purchased as pre-developed assays from Applied Biosystems and normalized to the expression of the *GUSB* housekeeping gene.

### Determination of differentially expressed target genes by real-time ready arrays

After RNA extraction, 500 ng of total RNA was used for cDNA synthesis using the Transcriptor High Fidelity cDNA Synthesis Kit (Roche Applied Science) according to the manufacturer's instructions. A 384-well custom array panel (Roche Applied Science) was designed to quantify the expression of genes involved in multiple cellular pathways, namely apoptosis, cell cycle, DNA repair and invasion by real-time PCR using a LightCycler 480 instrument (Roche Diagnostics, Basel, Switzerland). The relative expression of target genes was normalized using *GUSB, TFRC* and *18S* as housekeeping genes. The fold-variation in gene expression was determined using comparative Ct method and genes with fold change above 0.5 or below -0.5 were considered up or downregulated, respectively.

### Western Blot

Proteins were extracted from whole-cell lysates using RIPA lysis buffer (Santa Cruz Biotechnology, CA, USA) and subsequently quantified using a Pierce BCA assay (Thermo Scientific Inc., Bremen, Germany), according to the manufacturer's instructions. Briefly, 30 μg of protein from each sample were separated using 4–20% Mini-PROTEAN^®^ TGX^™^ Precast Gel at 120 V and subsequently blotted onto Protran nitrocellulose transfer membranes (Whatman, Dassel, Germany). For immunodetection, membranes were incubated overnight at 4ºC with antibodies directed against AcH3, AKT phosphorylated (S473), C-Jun phosphorylated (S73 and Thr91 (Millipore, Billerica, MA), Akt1, SRC (Santa Cruz Biotechnology, Santa Cruz, CA) EGFR phosphorylated (Y1172) (ProSci Inc., Poway, CA, USA) AR, DNMT1, p21 and PARP1 (Cell Signaling Technology, Danvers, MA) at 1/500, EFGR (Kinexus Inc., Vancouver, Canada), ERK1/2 (Enzo Life Sciences, Inc., Farmingdale, New York) and MEK1 (BD Biosciences, Franklin Lakes, NJ, USA) at 1/5000. The Immun-Star WesternC Chemiluminescent kit (Bio-Rad, Hercules, CA, USA) was used to develop the membranes which were then recorded with Amersham Hyperfilm (GE Healthcare Buckinghamshire, UK). To ascertain equal loading of protein, the membranes were incubated with a monoclonal mouse antibody against β-Actin (1:8,000, Sigma-Aldrich, CO., St. Louis, MO). Protein band intensities were determined using Quantity One software (Bio-Rad), by comparing the protein band intensity with the loading control (β-Actin).

### Quantitative Methylation Specific PCR (qMSP)

Genomic DNA was extracted from cell lines using a standard technique comprising digestion with proteinase K (20mg/mL) in the presence of 10% SDS at 55ºC, followed by phenol-chloroform extraction and precipitation with 100% ethanol. One microgram of DNA was submitted to bisulfite modification using the EZ DNA Methylation-Gold™ Kit (Zymo Research, Orange, CA) following the manufacturer's instructions. Bisulfite modified DNA was amplified by qMSP using TaqMan technology [42]. Specific *GSTP1, APC* and *RARβ2* primers and TaqMan probes were designed using the Methyl Primer Express Software v1.0 (Applied Biosystems). *β-actin* (*ACTB*) was used as an internal reference gene to normalize for DNA input and all qMSP reactions were performed as previously described [11]. Methylation levels for each sample were derived from calibration curves constructed using serial dilutions of bisulfite modified CpGenomeTM Universal Methylated DNA (Millipore, Billerica, MA). *GSTP1, APC* and *RAR2β* methylation levels were calculated after normalization for *ACTB*.

### Kinexus Antibody Array

The Kinex™ KAM-850 antibody microarray (Kinexus Inc., Vancouver, Canada) was used to perform an unbiased characterization of altered proteins after hydralazine exposure. In total, this microarray featured 854 antibodies of which 517 were pan-specific (for protein expression) and 337 were protein phospho-site-specific (for phosphorylation). Briefly, 50 μg of lysate protein from each sample were covalently labeled with a fluorescent dye and unincorporated dye molecules were removed by ultrafiltration. Purified proteins were then incubated on the antibody microarray. After the washing steps, the arrays were sent to Kinexus and were scanned using a Perkin-Elmer Scan Array Reader (Waltham, MA). Signal quantification was performed with ImaGene 8.0 (BioDiscovery, El Segundo, CA) with predetermined settings for spot segmentation and background correction. Z ratio analysis was used to identify differentially expressed proteins between the vehicle and hydralazine treated cells. Three independent replicates were performed for each condition. Functional annotation of the identified proteins was carried out with the online tool DAVID Bioinformatic Resources 2013 (version 6.7) (http://david.abcc.ncifcrf.gov/).

### EGFR Mutational Analysis

The EGFR tyrosine-kinase domain (exons from 18 to 21) was evaluated for activating mutations in LNCaP, 22Rv1, DU145 and PC-3 cell lines. High resolution melting (HRM) was used as a screening method, after which DNA sequencing was performed in all cell lines. HRM was performed on a LightCyclerW 480 II Real-Time System (Roche Diagnostics, Basel, Switzerland). PCR mastermix containing one primer pair, all PCR reagents, DNA and mineral oil was added to each well of a 96 well plate. Amplification and melting curves were generated and analyzed using the LightCyclerW 480 Gene Scanning software version 1.5 (Roche diagnostics, Basel, Switzerland.). The generated PCR amplification products were subsequently purified using illustra GFX PCR DNA and Gel Band Purification Kit (GE Healthcare Life Sciences, Little Chalfont, UK) according to the manufacturer's protocol. The sequencing reaction was performed using 1 μL of purified PCR amplification products, and Big Dye W Terminator V1.1 cycle sequencing Ready Reaction Mix (Applied Biosystems, Foster City, CA, USA). Sequencing PCR products were then purified in order to remove contaminants, using illustra SephadexW G-50 fine (GE Healthcare Life Sciences, Little Chalfont, UK) and run on an ABI PRISM™ 310 Genetic Analyzer. The obtained electropherograms were analyzed with Sequencing Analysis Software v5.2 (Applied Biosystems, Foster City, CA, USA) and read manually.

### Statistical analysis

One-way analysis of variance (ANOVA), with post-hoc Dunnet's multiple comparison test, when appropriate, was used to compare the results obtained in each parameter for the two different concentrations of hydralazine and the vehicle. Analysis was performed with SPSS software for Windows version 19.0 (IBM-SPSS Inc Chigaco, IL) and statistical significance was set at *p*<0.05.

## SUPPLEMENTARY MATERIAL FIGURES


