# Supplementary material for: Anti-neoplastic properties of hydralazine in prostate cancer
Source: Oncotarget. 2014 Apr 17;5(15):5950–64. doi: 10.18632/oncotarget.1909 (PMC4171604; doi:10.18632/oncotarget.1909)
Supplement: Supplementary file 1 [file oncotarget-05-5950-s001.pdf]

Anti-neoplastic properties of hydralazine in prostate cancer

Supplementary Material

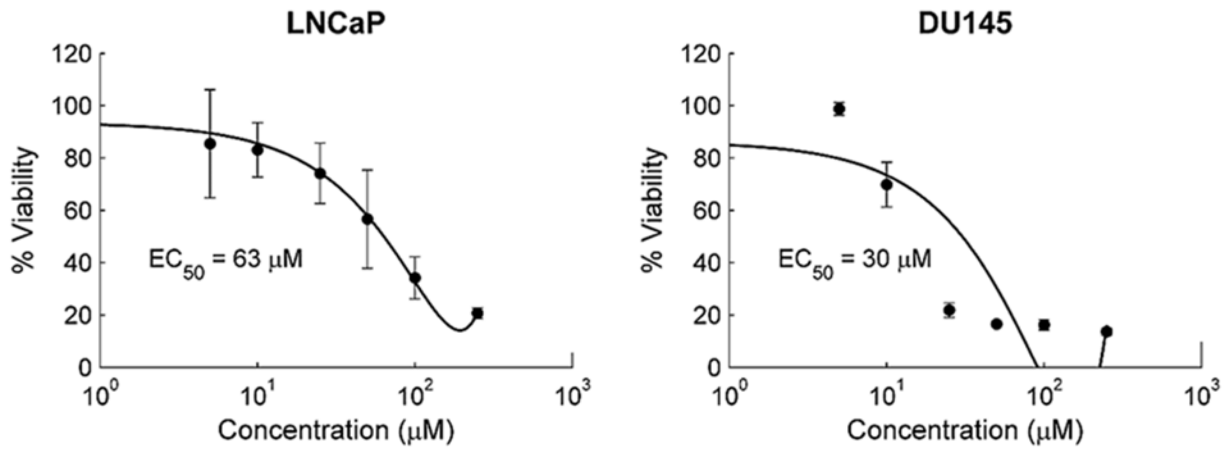

Supplementary Figure 1: Graphic representation of hydralazine half-maximal effective concentration (EC50) in LNCaP and DU145 cell lines, at 72h. LNCaP displayed an EC50 of 63 µM, whereas for DU145 only 30 µM were required.

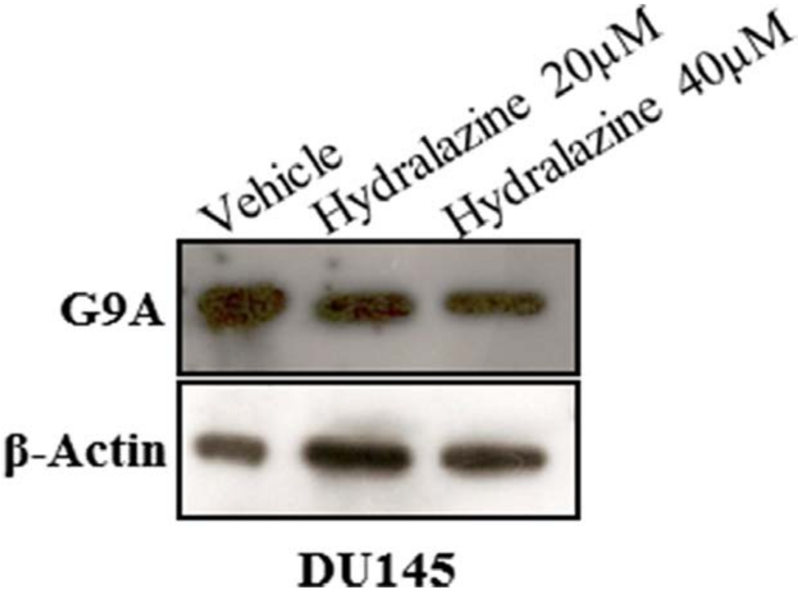

Supplementary Figure 2: Effect of hydralazine exposure on histone methyltransferase G9A expression. Western blot analysis of G9A, in DU145 cells, after 3 days of drug exposure. β-Actin was used as loading control.
